# In search of cost effective control of leptospirosis—a common zoonotic disease in developing countries

**DOI:** 10.2478/abm-2021-0019

**Published:** 2021-08-20

**Authors:** Editorial Office of Asian Biomedicine

**Affiliations:** Faculty of Medicine, Chulalongkorn University, Bangkok 10330, Thailand

Leptospirosis a result of infection by pathogenic spirochetes of the genus *Leptospira* and can affect both humans and animals. It is one of the most common neglected zoonotic diseases [1]. Leptospirosis occurs globally, with most clinical cases found in the tropics. *Leptospira* spp. infect various wild and domestic mammals. Reservoir mammals may shed the leptospires in their urine, contaminating the environment, particularly wet regions and water bodies [2]. Infections most often occur in humans after their exposure to environmental sources, such as water or soil contaminated with animal urine [2]. Leptospirosis is largely a water-borne disease in developing countries, resulting, in part, from the current global climate [3]. Surveillance systems vary considerably between countries and are often inadequate. Therefore, comprehensive information about the disease has not always been available [3].

The clinical course is variable and ranges from those who have a subclinical illness followed by seroconversion or a self-limited systemic infection to a severe, fatal illness with multiorgan failure [4]. Due to the nonspecific nature of clinical features, many patients present with acute febrile illnesses and tests need to be planned to arrive at a probable diagnosis [5]. In a review of diagnosis of acute febrile illnesses, the use of 5–22 tests per patient was needed to evaluate acute undifferentiated febrile illness with nonspecific symptoms. Among possible causes include malaria, dengue, scrub typhus, enteric fever, and leptospirosis [5].

There is high demand for suitable point-of-care diagnostic tests for leptospirosis because its incidence varies between geographic areas [5, 6]. Confirmation of the disease includes not only the most widely used reference standard method of a microscopic agglutination test for antibody detection, but also direct microscopic demonstration and culture to isolate leptospires [7]. However, high costs, poor standardization, elaborate sample preparation, or all of these factors prevent routine use at the point of care [7]. Therefore, in most clinical settings, health care practitioners have to depend on insensitive and less-specific serological methods, which dominate the diagnostic landscape in the developing world [7]. Serology is nevertheless an important tool for a quick diagnosis of leptospirosis so that appropriate therapy can be administered [6]. However, there is an urgent need for improvement toward greater compliance with some of the point-of-care criteria. Loop-mediated isothermal amplification (LAMP), a method for amplifying DNA, could be useful for diagnosis of leptospirosis during the first week of illness before substantial numbers of antibodies are generated, whereas an IgM enzyme-linked immunoassay (ELISA) could be used as the mainstay of diagnosis from the second week onward. Further studies, especially those community based, comparing ELISAs, polymerase chain reaction (PCR), LAMP, culture isolation, and microscopic agglutination tests are required to evaluate the veracity of this recommendation [8].

Control of leptospirosis requires a One Health approach, and improvement of diagnostic tools to detect *Leptospira* spp. in animals is as important as it is in humans. In this August 2021 issue, Lee et al. [9] report the use of a LAMP system using sucrose as a stabilizer to be used for field surveil-lance and epidemiological study, particularly in resource-limited settings. A previous study indicated that lyophilized LAMP reagents are stable for 3 months when stored at 4 °C and 1 month when stored at 25 °C [10]. The addition of sucrose attempts to address the limitations of cold storage requirements for LAMP reagents and use of lyophilization equipment. The LAMP reagent mixture with sucrose can maintain *Bst* DNA polymerase activity at room temperature for at least 45 days. The findings of this study will contribute to the design of room-temperature-stable premixed LAMP reagents, making LAMP an ideal tool for the rapid detection of leptospiral bacteria for diagnosis, field surveillance, and epidemiology in resource-limited settings [9].

However, the new premixed LAMP reagents with sucrose stabilizer should be compared with criterion standards of diagnosis of leptospirosis in a blinded and independent fashion. Currently, the best reference standards for diagnosis of leptospirosis are the microscopic agglutination test and isolation by culture, although both are acknowledged as imperfect, either alone or in combination [11]. Nevertheless, the result of a comparison could render more confidence in the application of LAMP as a point-of-care technique in our quest for cost effective control of this important zoonotic infection in resource-limited countries.
